# Attention Cost-Sensitive Deep Learning-Based Approach for Skin Cancer Detection and Classification

**DOI:** 10.3390/cancers14235872

**Published:** 2022-11-29

**Authors:** Vinayakumar Ravi

**Affiliations:** Center for Artificial Intelligence, Prince Mohammad Bin Fahd University, Khobar 34754, Saudi Arabia; vravi@pmu.edu.sa

**Keywords:** skin disease, deep learning, transfer learning, attention, cost-sensitive, meta-classifier

## Abstract

**Simple Summary:**

According to skin disease reports by healthcare organizations, the number of cases of skin disease is growing gradually over the years globally. In skin disease diagnosis, dermatologists examine skin cells by using a dermatoscope. Due to the global shortage of expert dermatologists, mainly in developing countries, an accurate early skin disease diagnosis is not possible. To automate the examination of skin disease images, computer-aided diagnosis-based tools are used in healthcare and medical environments. Computer-aided diagnosis employs machine learning including deep learning models on skin disease images to detect and classify skin diseases. The present work proposes a deep learning-based model to accurately detect skin diseases and classify them into a family of skin diseases using skin disease images. The proposed system demonstrated a performance improvement of 4% accuracy for skin disease detection and 9% accuracy for skin disease classification compared to the existing deep learning-based models. The proposed computer-aided tool can be used as an early skin diagnosis tool to assist dermatologists in healthcare and medical environments.

**Abstract:**

Deep learning-based models have been employed for the detection and classification of skin diseases through medical imaging. However, deep learning-based models are not effective for rare skin disease detection and classification. This is mainly due to the reason that rare skin disease has very a smaller number of data samples. Thus, the dataset will be highly imbalanced, and due to the bias in learning, most of the models give better performances. The deep learning models are not effective in detecting the affected tiny portions of skin disease in the overall regions of the image. This paper presents an attention-cost-sensitive deep learning-based feature fusion ensemble meta-classifier approach for skin cancer detection and classification. Cost weights are included in the deep learning models to handle the data imbalance during training. To effectively learn the optimal features from the affected tiny portions of skin image samples, attention is integrated into the deep learning models. The features from the finetuned models are extracted and the dimensionality of the features was further reduced by using a kernel-based principal component (KPCA) analysis. The reduced features of the deep learning-based finetuned models are fused and passed into ensemble meta-classifiers for skin disease detection and classification. The ensemble meta-classifier is a two-stage model. The first stage performs the prediction of skin disease and the second stage performs the classification by considering the prediction of the first stage as features. Detailed analysis of the proposed approach is demonstrated for both skin disease detection and skin disease classification. The proposed approach demonstrated an accuracy of 99% on skin disease detection and 99% on skin disease classification. In all the experimental settings, the proposed approach outperformed the existing methods and demonstrated a performance improvement of 4% accuracy for skin disease detection and 9% accuracy for skin disease classification. The proposed approach can be used as a computer-aided diagnosis (CAD) tool for the early diagnosis of skin cancer detection and classification in healthcare and medical environments. The tool can accurately detect skin diseases and classify the skin disease into their skin disease family.

## 1. Introduction

In the present era, skin diseases are one of the leading infectious diseases among people globally. Skin diseases are common in fair-skinned populations. Skin diseases can be permanent or temporary and these are painless or painful. The number of cases of skin cancer has been high in the recent year in the United States and Australia [1]. In addition, the total cost involved in the treatment of skin diagnosis has been high compared to other cancers and this was reported by the government of Australia and the United States. A report by the Skin Cancer Foundation shows that the number of skin disease cases continues to increase worldwide in the future [2,3]. Dermoscopy is a non-invasive imaging technology that can examine skin lesions with a dermatoscope. This technology removes the surface reflection of the skin and obtains more informative visual information by going into deeper levels of the skin. This type of technology has enhanced the diagnosis of skin cancer detection and classification. In developing countries and in the world, the number of dermatologists is not sufficient, as skin diseases become high every year. Moreover, dermatologists need to be experts with good experience in achieving good accuracy otherwise the performance of dermatologists in accurately detecting skin disease will not be high [2]. There may be a possibility that the appearance of multiple skin diseases is similar and expert dermatologists’ accuracy on similar multiple skin disease diagnoses will not be high.

To automate the diagnosis of skin lesion data samples, CAD tools were introduced [4]. CAD tools can be used for an early skin disease diagnosis. In the development of CAD-based tools, to automate the process of skin disease detection and classification, researchers employed various data mining and machine learning algorithms on the images of skin diseases [5]. Various feature engineering and feature selection approaches were investigated to accurately detect skin cancers by passing the features into various machine learning and data mining algorithms. The survey of skin disease detection and classification shows that there are various studies based on supervised, semi-supervised, and unsupervised approaches [3]. The performance of supervised-based methods is high compared to the semi-supervised and unsupervised approaches [6]. Thus, the current study considered the supervised-based approach to accurately detect skin diseases and classify them into their skin disease family. The major issue that exists in data mining and machine learning-based skin disease detection and classification is that the model’s performance relies on optimal features [7]. These features are extracted manually and require a domain-level knowledge of image processing and skin diseases. This type of feature engineering and feature selection process is not easy. This may require more cost, and time complexity will be high. Most importantly, the attacker can compromise the CAD-based system if the features are known using the concepts available in the domain of adversarial machine learning. So, the machine learning-based CAD approach for skin disease detection and classification may not be completely considered robust in an adversarial environment, since the current healthcare system is connected to the internet and the healthcare networks and their connected devices are open to attacks. In addition to the performance of the model, the security of the model for skin disease detection and classification is important in the healthcare environment. In addition to the robustness of the model, the generalization of the model is important, i.e., there may be a possibility that the machine learning-based model may not work well for new skin diseases or the variants of the existing skin disease detection and classification.

A recent literature survey demonstrates that deep learning-based approaches were employed for skin disease detection and classification [8]. The deep learning-based approaches outperformed machine learning and data mining-based approaches in skin disease detection and classification, using samples of skin images. Various studies reported that the performance of deep learning is higher compared to the data mining and machine learning-based studies on benchmark data sets, i.e., the International Skin Imaging Collaboration (ISIC) archive. The studies have finetuned ImageNet-based pretrained models for skin disease detection and classification and reported that the finetuned model performances are higher compared to the non-finetuned models. In addition to that, the pretrained models require less time compared to the non-pretrained models. This is mainly due to the reason that the ImageNet-based pretrained models were trained with very big databases of natural images with several different classes. Though the medical images are different compared to natural images, the weights learned on natural images are finetuned with the medical images. This type of finetuned model shows better performances in various medical image classification tasks including skin disease detection and classification. The existing studies for skin disease classification using deep learning-based pretrained models used SoftMax at the last layer with a fully connected network for classification [3]. However, instead of using SoftMax, the features of the finetuned models can be further passed into other classical machine learning classifiers and this type of approach can be called a large-scale machine learning classifier. This type of classifier has the capability to show better performances compared to the SoftMax with a fully connected network. In addition to using one pretrained model for skin disease detection and classification, an ensemble of pretrained models can be employed. Since each pretrained model has the capability to extract its own features, there may be a possibility that ensemble-based models can enhance the performance of individual models. The survey shows that this type of ensemble approach demonstrates a better performance compared to the individual classifiers [5]. The classes in the benchmark datasets of skin diseases are highly imbalanced and most of the existing models for skin disease classification are not effective in handling rare skin diseases. In order to handle the data imbalance, authors have used data augmentation and Generative Adversarial Network (GAN)-based approaches. However, these are not effective in handling the data imbalance of skin diseases and though the studies reported good performances, there may be a possibility that the models may not perform well on the datasets that are from different modalities or patients from different regions. The proposed work’s major contributions are given below

- The current work proposed an attention-cost-sensitive deep learning-based feature fusion ensemble meta-classifier approach for skin cancer detection and classification.
- Detailed investigation and analysis of convolutional neural network (CNN)-based pretrained model for skin disease detection and classification.
- Fusion of features from CNN-based pretrained models is proposed to enhance the performance for skin disease detection and skin disease classification.
- Attention is integrated into the CNN-based pretrained model to extract the optimal features to accurately detect skin diseases.
- Cost-weights are introduced during the training of a model to handle data imbalance in the skin disease dataset.
- To improve the performance of the SoftMax-based fully connected network classifiers, a two-stage classification model is proposed.
- Comparison of the proposed model with other CNN-based pretrained models and other existing studies.

The remaining parts of the paper are organized as follows. The literature survey of skin disease detection and classification is included in Section 2. Detailed information on the proposed method is discussed in Section 3. The description of datasets is included in Section 4 and statistical metrics are included in Section 5. Results and discussion of the proposed approach for skin disease detection and classification are included in Section 6. Finally, the conclusion and feature works are included in Section 7.

## 2. Literature Survey

Skin disease detection and its classification is a long-standing problem in the field of artificial intelligence. Prior to deep learning, various feature engineering methods were employed on the skin disease image database and further various classical machine learning algorithms were employed for skin disease detection and its skin disease family classification. However, with the recent surge of deep learning methods in performance improvement in various applications in medical imaging, the literature survey shows that methods of deep learning have been employed for skin disease identification and skin disease classification. The detailed literature survey summary of the existing methods for skin disease detection, skin disease classification, and available datasets for skin disease is discussed in detail by authors [2,3,5,8]. The literature survey shows that deep learning and artificial intelligence-based approaches outperform dermatologists’ performance in accurately detecting skin diseases and classifying them into their skin disease family. However, there are various scenarios in which the best-performed model in the existing survey may not perform well. One such case study is rare skin diseases or skin diseases that have a smaller number of data samples. In addition to this, the performance of the models can be enhanced by adding clinical features. In this literature survey section, the existing works summary and its main limitations are discussed by comparing it with the proposed work.

The GoogleNet Inception v3 CNN architecture was employed for skin disease classification and its performances were evaluated against dermatologists against different test cases [9]. However, the model performances were not evaluated in detail and the models are not robust and generalizable. The ResNet50-based model was proposed for skin disease classification [10]. The model performances were assessed in different test cases with the involvement of healthcare and dermatologists. Though the proposed model achieved better performances compared to the existing methods, the models cannot be employed in a real-time environment, and in addition, this study cannot be considered for benchmarking the machine learning and deep learning models. The reason is the dataset was collected from different publically available sources and there may be overlapping of samples in training, validation, and testing datasets. This is one of the reasons the study reported good performances in all the test cases. Multichannel CNN with Gabor wavelet-based approach is proposed for skin disease classification [11]. The authors reported the performance of the proposed model by using ISIC 2017 datasets. Since the dataset is highly imbalanced, the proposed method’s detailed study of handling rare skin diseases is required. Without this, the proposed method may not be considered robust for skin disease classification. In addition to that, the authors have considered only a smaller number of classes from the dataset, mainly the performance of the study demonstrated for melanoma skin disease. A hybrid of classical image processing feature engineering, clinical features, and automated feature engineering using ResNet-50 models is proposed for skin disease classification [12]. The performances of the proposed study were demonstrated on the datasets NIH SBIR dermoscopy studies and ISIC 2018. Though the model reported better performances on both datasets, the authors did not show the performance of the proposed model in handling rare skin diseases.

To develop a generalized skin disease classification model, the authors adopted the domain adaptation deep learning approach using CycleGAN and its performance shown on the HAM10000 dataset [13]. The proposed model is generalized, and its experiments and results reported by the authors show that the model was able to classify the skin disease samples by handling different cohorts with different shifts. The patch-based attention approach is proposed for skin disease classification [14]. To handle the data imbalance after patching the skin disease samples, the proposed approach uses various cost-sensitive approaches. The authors report that the proposed approach performs better than the existing methods and handles data imbalances during training. The performance of the methods was shown on more than one skin disease dataset, including the HAM10000 dataset. CNN-based approach with a novel optimizer-based approach is proposed for skin disease classification using the ISIC skin disease dataset [15]. However, the authors did not demonstrate a detailed analysis of the proposed model to identify the robustness and generalization to accurately detect skin diseases. In [16], the authors used the support of binary classification and enhanced the performance of the GoogLeNet and Inception-v3 model by 7% in skin disease classification with seven classes. The proposed model is not effective in an imbalanced skin disease database, and in addition, the model is not effective in extracting the tiny portions of the infected region from the overall skin image. To increase the number of data samples for rare skin diseases, StyleGANs approach is employed. Later, the authors developed a method for skin disease classification by fusing the CNN-based pretrained models. Though the proposed method alleviates the data samples for rare skin diseases, the proposed StyleGANs may not generate samples that are similar to the samples collected from the patients in real time. This type of GAN-based approach may not be considered accurate to handle rare skin diseases.

A fusion of handcrafted features and automated features from a CNN-based deep learning model was used in accurately detecting skin diseases [17]. Since the proposed model depends on hand-crafted feature engineering, the model time complexity is high and this type of approach may not be appropriate in a real-time skin-disease classification system in the healthcare environment. This is because deep learning itself can identify the optimal features and in addition to this, segmentation using deep learning can be incorporated. A two-stage approach was proposed for skin disease classification [18]. The first stage does the segmentation and classification of the segmented data classified in the classification stage. The authors employed fully resolution CNN in the first stage for segmentation and Inception-v3, ResNet-50, Inception-ResNet-v2, and DenseNet-201 for classification. The authors have conducted a detailed study using ISIC 2016, 2017, and 2018 datasets. The rare disease in ISIC datasets is handled using augmentation. However, the data augmentation approach may not be the right approach to handle the data imbalance and the literature survey on data augmentation shows that it cannot improve the performance of rare skin diseases. To handle various image sizes of skin disease, the authors have proposed a multiscale model with an ensemble of more than one CNN-based pretrained model for skin disease classification [19]. The proposed approach performances are demonstrated on the ISIC 2017 and 2018 datasets. Since the proposed approach is cost-insensitive, the model performance may not be considered good in the rare skin disease classification. With the aim to handle rare skin diseases in the ISIC 2018 dataset, the authors proposed a GAN-based approach for data augmentation and CNN for classification [20]. The authors reported that the GAN-based data augmentation approach with CNN performed better than the CNN. Data augmentation is not the right approach to handle the imbalance in the skin disease data sets. It may be possible that the GAN-based generated images are not entirely new samples and there will be a bias in learning.

The authors reported 7% improvement in accuracy by using clinical information along with a skin disease image database [21]. This database is a privately collected dataset using a phone camera. The dataset is balanced and moreover, there may be bias in training and testing datasets. Since the authors have not demonstrated the datasets of training and testing collected in different healthcare environments with different patients, the proposed approach may not be considered robust for skin disease classification. An ensemble of various pretrained model performances was shown for skin disease classification using the ISIC 2018 dataset [22]. The proposed model may not be effective in achieving good performance in classifying the rare disease as the proposed method is not giving any kind of importance to the minor classes of skin disease during the training of an ensemble model. To detect skin disease accurately, segmentation was employed before the classification [23]. The authors compared the proposed segmentation approach performance with U-Net, and in all the test cases, the proposed approach demonstrated better performances. The performance of CNN and other classical machine learning models’ performances were demonstrated for classification. With several test cases, authors have demonstrated that the proposed model shows better performances compared to the existing approaches using the ISIC 2018 skin disease dataset. Though the proposed model is robust in accurately detecting skin diseases, the authors did not show the proposed method’s performance in handling rare skin diseases. A fusion of CNN-based pretrained models was proposed for skin disease classification [24]. The performance of the proposed models was evaluated on the ISIC 2016 dataset. The authors report that the fused model demonstrates better performances in detecting skin disease compared to the non-fused and existing studies. However, the detailed performance of the proposed study is not evaluated on ISIC 2016. Since the dataset is highly imbalanced, it may be possible that the bias existed during learning a skin disease model in training. The ResNeXt101-based model is evaluated for multi-class skin disease classification using the HAM10000 dataset [25]. The authors reported that the proposed model performed better than the non-pretrained and pretrained models. However, the detailed performance of the proposed model is not shown for handling rare skin diseases.

A 34-layer residual network-based approach is proposed for skin disease classification using the HAM10000 dataset [26]. The performance of the proposed approach is evaluated in various clinical settings and the authors reported that the proposed approach performs better than the existing methods, and in some test cases outperforms the dermatologists. However, the robustness and generalizability of the proposed approach are not shown in detail for skin disease classification. A decision fusion of GoogleNet, ResNet-101, and NasNet-Large is proposed and its performances are shown on the skin disease classification using the ISIC 2019 dataset [27]. The proposed method demonstrated better performances compared to the related existing methods. However, the detailed evaluation and analysis of the proposed method are not evaluated on rare skin disease classification. The inception-v4-based model was proposed for skin disease classification [28]. The model uses a hybrid of clinical features and images of skin disease and classifies the patient skin samples into 27 classes. The model performance on rare skin diseases is required to understand the robustness of the proposed method in handling the imbalanced data set of skin diseases. ResNet152 and InceptionResNet-V2 with a triplet loss-based approach were proposed for identifying the skin disease and their performance was shown on a publically available dataset [29]. The authors demonstrate that the method performed well compared to the other methods, however, the detailed performance of the method is not demonstrated for rare skin diseases or the minority classes of skin diseases. CNN-based model is proposed for skin disease classification. The proposed model supports the multi-class skin disease classification [30]. The performance of the proposed model is shown on the datasets of ISIC-17, ISIC-18, and ISIC-19. These three datasets are well-known datasets and are used for benchmarking the models of machine learning and deep learning in detecting skin disease and classifying the detected skin disease to its skin disease family. All of these three datasets are highly imbalanced, such as, some skin disease are rare, and contain a smaller number of data samples. This may be one of the reasons that the proposed method reports good performances even by using a non-pretrained CNN model. To handle rare skin diseases, an attention-based GAN deep learning approach is proposed [31]. The authors demonstrate that the proposed method generates skin disease samples that are from different distributions and it is considered to be more effective than data augmentation. However, even though the attention-based GAN has the capability to generate skin disease samples from different distributions, the generated sample may not be the same as the data samples collected from patients in real time.

The authors propose a three-stage approach for skin disease classification [32]. The first stage employs MaskRCNN for segmentation and feature extraction using DenseNet in the second stage and classification using a support vector machine (SVM). The proposed model performances are demonstrated on the datasets of ISBI2016, ISBI2017, and HAM10000. The experiments reported in the paper demonstrate that the proposed model achieves better performances compared to the existing models. The proposed model is computationally expensive and in addition, the proposed model performances are not shown in detail for rare skin diseases. DenseNet201 network-based approach is proposed for skin disease classification using HAM10000 dataset [33]. The proposed method demonstrated better performances compared to the existing non-pretrained models. The authors did not show detailed experiments on the generalization and robustness of the proposed method in skin disease classification. A hybrid of MobileNet V2 and the long short-term memory (LSTM)-based approach is proposed for skin disease classification [34]. This method has outperformed the existing methods by showing more than 85% accuracy on the HAM10000 dataset. The robustness and generalizability of the proposed method for skin disease classification are not discussed in detail. The authors have demonstrated that the CNN-based model performance is similar to the performance obtained from clinical experts [35]. However, the authors did not show a detailed analysis of the proposed approach in different experimental settings. Thus, the proposed method cannot be considered robust and accurate. The summary of the existing works on skin disease detection and skin disease classification is summarized in Table 1.

The detailed literature survey of the aforementioned works shows that ImageNet-based pretrained models are employed for skin disease detection and its family classification with the aim to enhance the performance of non-pretrained models. However, the existing studies on skin disease databases demonstrate that the available standard database in the literature is highly imbalanced, and also, some skin diseases are rare. As a result, rare skin diseases contain very a much smaller number of data samples. Thus, the existing models are not accurate in predicting this rare skin disease and the existing models are highly dominant to the skin disease that are common and have a very high number of data samples. In a skin image database, the disease is a tiny portion of the overall skin image and it may be possible that the existing study might miss this type of important tiny region in accurately identifying the skin disease. Even though the CNN-based models have the capability to extract the important regions from the skin disease image, extraction of important and optimal features from the infected region in the overall image is limited by the CNN-based pretrained models. In addition, each CNN-based pretrained models have the capability to extract its own features to accurately identify the skin disease and classify the skin disease to its family. The features are unique and disjoint from each other. In the proposed work, cost-sensitive learning is introduced to the CNN-based pretrained model to avoid bias in learning during training, and equal importance is given to all the classes of skin disease. The various EfficientNetV2-based pretrained models were extracted and further, the dimension of the feature was reduced using the dimensionality reduction approach, i.e., PCA. Further, the features are combined and passed into the meta-classifier for skin disease detection and skin disease classification. The stacked classifier is a two-level approach; the first level includes the random forest (RFTree) and SVM for the prediction of skin disease, and later these predictions were classified accurately using the logistic regression in the second level.

## 3. Proposed Methodology for Skin Cancer Detection and Classification

**Figure 1:**
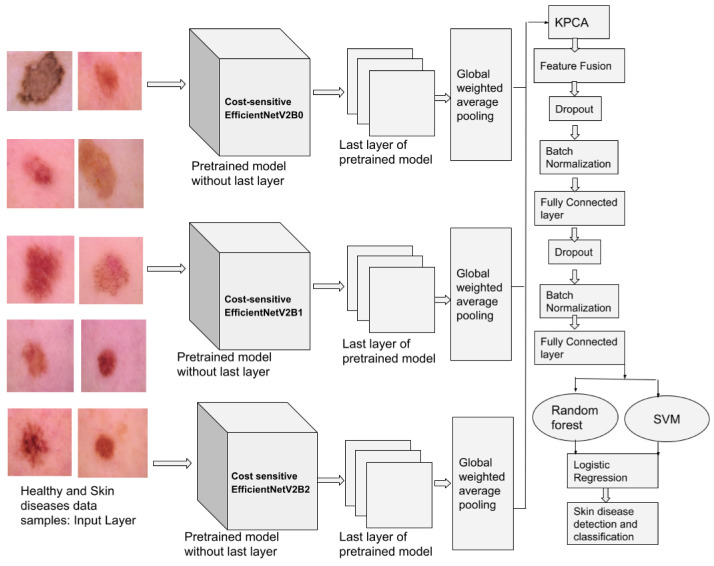
Proposed methodology for skin disease detection and skin disease classification.

The proposed methodology for skin disease detection and skin disease classification is shown in Figure 1. The details of the proposed architecture are given below.

The skin images of patients are preprocessed in the input layer. The preprocessing includes transforming the dimension of the image into input dimensions of the CNN-based pretrained model. After reading the image data, the data are transformed into the [0–1] range by applying normalization.

The existing literature survey shows that the CNN-based pretrained models have been employed for skin disease detection and classification. The pretrained models of the ImageNet database are finetuned on the skin image database. This type of finetuned model has demonstrated better performances compared to the non-finetuned models. This work employs various CNN-based pretrained models for skin disease detection and classification. The pretrained models considered in this work are Xception, VGG16, MobileNet, ResNet50, InceptionV3, DenseNet121, EfficientNetB0, EfficientNetV2B0, EfficientNetV2B1, and EfficientNetV2B2. All these models have an input layer, more than one hidden layer, and a classification layer. In the hidden layer, the pretrained models contain more than one convolution layer, pooling layer, and fully connected layers. Between the convolution and fully connected layers, the model contains batch normalization and dropout layers.

The current work employs EfficientNetV2 model for skin disease detection and classification. EfficientNetV2 model is a pretrained model on the ImageNet database. This database contains 1000 classes of natural images and the models have learned a rich feature representation by training a model using a very big image database. In this work, the EfficientNetV2 pretrained model is finetuned on skin disease detection with two classes by replacing the last layer of the EfficientNetV2 pretrained model and skin disease classification with seven classes by replacing the last layer of the EfficientNetV2 pretrained model.

**Figure 2:**
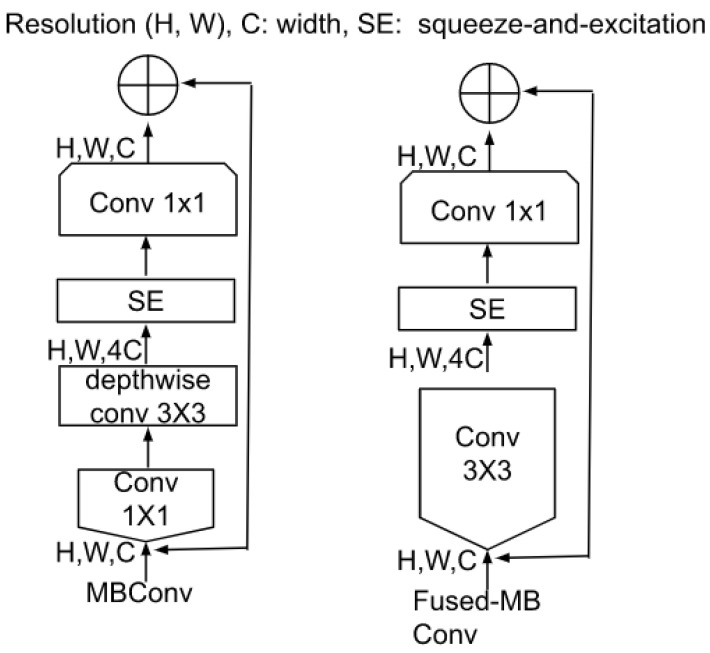
Architecture of MBConv and Fused-MBConv.

Recent years’ work demonstrates that many experiments carried out by researchers find out the efficient CNN architecture in deep learning. The architecture maintains a balance among accuracy, speed, FLOPs, etc. For example, to improve the performance of the model with better accuracy, DenseNet and EfficientNet model were introduced. The same authors of EfficienNet architecture studied the limitations of the EfficienetNet architecture and developed a new architecture called EfficientNetV2. EfficientNetV2 is a family of models such as EfficientNetV2B0, EfficientNetV2B1, EfficientNetV2B2, EfficientNetV2B3, EfficientNetV2S, EfficientNetV2M, and EfficientNetV2L. In EfficientNetV2 architecture, the authors developed techniques to improve the model performances with a smaller number of parameters and improve the model inference time. The authors included the following techniques in EfficientNetV2:

- Neural architecture search (NAS): To find optimal parameters and model design, the authors employ random search and reinforcement learning techniques.
- Scaling: Authors have employed the compound scaling rule of the EfficientNet model. However, the modification was conducted to the compound scaling scheme to avoid memory issues due to the increase in the size of the image.
- Training: Authors employ new regularization methods, and training model guidelines to improve the efficiency during training of an EfficeienNetV2 model.
- Progressive learning: The training of the model is accelerated by progressively increasing the size of the image.
- Convolutions and their building blocks: The EfficientNetV2 models use various types of convolutions, mainly Fused-MB Conv instead of MB Conv. The detailed architecture information of Fused-MB Conv and MB Conv is shown in Figure 2.

The above-modified methods make the EfficientNetV2 model achieve better performance by increasing the speed during training a model compared to EfficientNet models ranging from B0 to B7.

Since the CNN-based pretrained models are ineffective in handling the imbalanced datasets, the current work integrates a cost-sensitive learning approach for CNN-based pretrained models. During backpropogation, the current work follows an algorithmic approach to include the misclassification costs to handle the bias in the training of a model. The skin disease sample of patient *S* is connected with a cost item [C[class(S),t], where class(S) and *t* are the actual and predicted class, respectively. The current work assigns less cost to the classes that contain more data samples and high cost to the classes that contain a lesser number of skin disease data samples. Since the values of the cost matrix are empty at the beginning, the current work follows the Gaussian distribution to assign the values for the cost matrix. These values in the cost matrix are finetuned across epochs. The loss function for cost-insensitive CNN-based pretrained models is defined as

(1) $$J=-\sum_{s\in samples}\sum_{n}t^{n}\log pred^{n}$$

The loss function for the cost-sensitive model is given below:

(2) $$J=-\sum_{s\in samples}\sum_{n}t^{n}\log pred^{n}C\left[class\left(s\right),n\right]$$

where *S* is a loss function, $pred^{n}$ denotes the predicted output of the nth output neuron, $t^{n}$ is the target value, C[class(s),n] denotes the cost with class(s) and is the exact value of sample *s*, and *n* is the predicted class of sample *s*.

The EfficientNetV2 models such as EfficientNetV2B0, EfficientNetV2B1, and EfficientNetV2B2 networks contain more than one convolutional layer and pooling layers followed by a series of fully connected layers. There may be a possibility that passing the high-dimensional feature representation learned from the series of a convolutional and pooling layer to a fully connected layer results in overfitting and hinders the model’s generalization ability. Since more than one fully connected layer is involved at the end of the networks before classification, dropout and regularization layers are required. There may be a possibility that the dropout and regularization layers result in the loss of important features. To overcome the series of fully connected layers with dropout and regularization layers, in this work, instead of passing the extracted features from finetuned model to global average pooling, the current work sends the extracted features to an attention layer. Global average pooling is a simple approach that estimates the average output of each feature map in the previous layer. Since some of the features are more important than others in each feature map of the previous layer, an attention mechanism is introduced that turns pixels in the GAP on or off. Next, rescale results on the number of pixels. The attention approach employed in this work is similar to global weighted average pooling. Since the dimensionality of the features of the finetuned model is high, KPCA is used. This helps to reduce the dimension of the features. KPCA is an improved version of PCA. It employs a kernel that allows projecting the data onto a higher dimensional space where the data points become linearly separable. Though there are many types of kernels available, this work employs the Radial basis function (rbf). This is mainly due to the reason that the data samples of skin disease are highly non-linearly separable. This is mainly due to the reason that the skin disease is very similar to each other. There may be a possibility that other kernels might perform better than the rbf kernel. Thus, a detailed analysis of the importance of kernel and other hyperparameters of KPCA will be considered as future work. The reduced feature representation of EfficientNetV0, EfficientNetV1, and EfficientNetV2 are fused. Since there are many advanced feature fusion methods available in the literature, employing them in the current work to learn better feature representation to accurately detect skin disease and classify them into their skin disease family will be considered as one of the significant directions toward future work. Further, the reduced features were passed into the ensemble meta-classifier.

The meta-classifier is a two-stage classifier, the first stage contains SVM and RFTree for prediction and the second stage contains the logistic regression for classification. SVM is a kernel-based machine learning algorithm used for solving problems related to classification and regression. SVM considers each data point in skin disease data samples in an n-dimensional plane and partitions them into two classes. The hyperplane line is selected based on the maximum margin among the two classes’ data points to distinguish the two classes. The selection of the kernel plays an important role in achieving good performance. The most commonly used kernels are rbf, linear, and poly. RFTree randomly constructs multiple decision trees and applies the input datasets. The output classification of each decision tree is considered to perform the ensemble learning to determine the final output. One of the well-known ensemble methods used in classification is the maximum number of RFTree trees voted for any particular class, considered as the outcome of the given input. Logistic regression is the probability modeling of the outcome given an input variable. Logical regression can be used for solving binary or multi-class problems. The logistic function will be a Sigmoid function taking any input value and classifying it as 0 or 1 for the binary classification of skin diseases. These machine learning classifiers are used in our framework to perform the ensemble meta-classifier-based feature fusion skin disease detection and classification. The SVM, RFTree, and logistic regression classifiers have parameters. The optimal performance depends on the parameters. This work has run several trials of experiments to identify the best parameters for SVM, RFTree, and logistic regression. The best parameters for SVM are tolerance = 0.0001, max iter = 5000, kernel = linear, and regularization parameter C = 1.0. The important parameters and the values of RFTree are n_estimators = 100 and max depth = 200. For both SVM and RFTree, random_state is set to 50. In logistic regression, tolerance, max_iter, and c is set to 0.0001, 100, and 1.0, respectively.

The steps involved in skin disease detection and skin disease classification using the proposed model are shown in Algorithm 1. Skin disease and Healthy samples are inputs to the proposed model and after that, the images are passed through several hidden layers to extract the features to recognize skin diseases. Finally, the proposed model outputs the label for the skin samples as either Healthy or Malignant in skin disease detection, and in the case of skin disease classification, the skin samples are classified into corresponding skin disease families.
**Algorithm 1:** Skin disease detection and classification.     
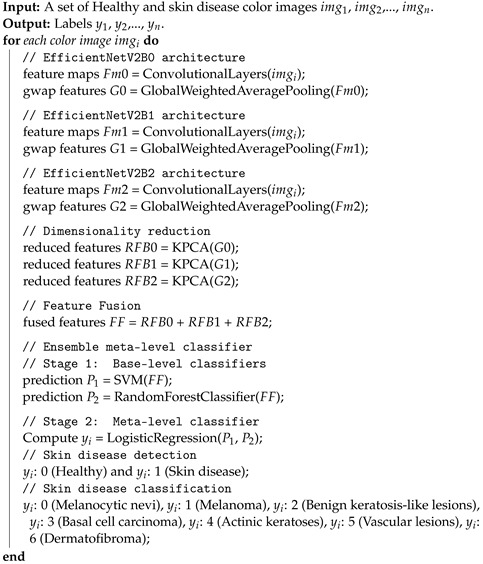


The proposed model takes skin disease image samples as input and outputs a value as either skin disease or healthy. Further, the model classifies the detected skin disease into its skin disease family.

## 4. Description of Datasets

The detailed statistics of skin diseases databases used in skin disease detection and skin disease classification are provided in Table 2 and Table 3, respectively. The data statistics show that the datasets are highly imbalanced. Without the proper handling of this type of dataset during training a model, there may be a possibility that due to bias, the models demonstrate better accuracy. The models might not learn better feature representation of the minority classes of skin diseases. To avoid this, the current work assigns the higher cost weights for the minority classes and lower-cost weights for the majority classes of skin disease during training a model. This type of assignment of cost-weights during training a model helps to avoid bias and gives importance to all skin diseases.

**Figure 3:**
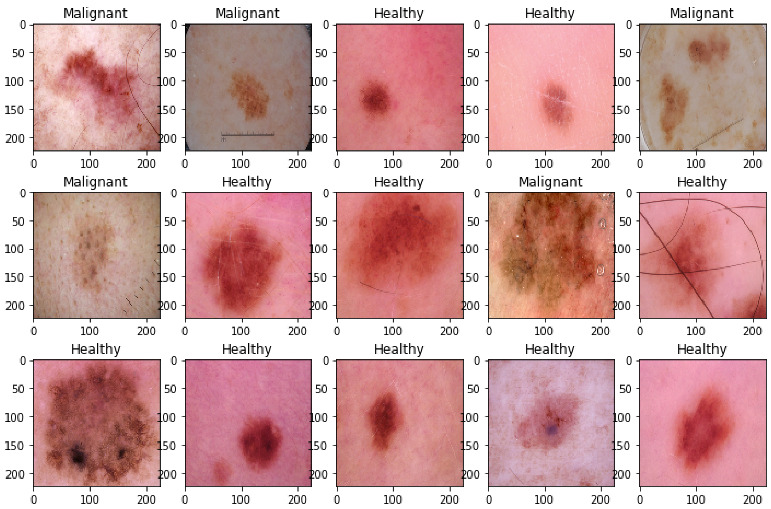
Skin image samples of healthy and malignant from skin disease dataset.

Skin disease samples of Healthy and Malignant are shown in Figure 3. These images are randomly chosen from the skin disease detection dataset. The images for skin disease detection are taken from the publicly available data repository, the ISIC archive. The images shown in Figure 3 demonstrate that the skin samples, both healthy and malignant, have higher intra-class and inter-class similarity. Since most of the samples of healthy and malignant look similar, there may be a chance that misclassification can be performed by the dermatologists. The chances of misclassification rate are very high. To avoid this, this work proposed a CAD-based tool by using an advanced deep learning approach with meta-classifier learning that extract the optimal features to accurately discriminate between the healthy and malignant.

A benchmark dataset for the development of CAD-based tools provided by the ISIC. The HAM10000 (“Human Against Machine with 10000 training images”) skin disease dataset is publicly available in the ISIC archive. The HAM10000 dataset is considered to be one of the well-known datasets and it is used in many studies to benchmark the machine learning and deep learning models for skin disease detection and skin disease classification. This dataset was collected from different populations with different modalities. Patients are from both male and female groups and most patients’ ages are in the range of 30 to 44. The data of skin disease classification shows that the skin disease increases with the increase in the age. Skin diseases are less for children aged less than 10. Skin diseases are most prominent if males are compared to females according to the skin disease classification dataset i.e., HAM10000. The most found skin disease among people is melanocytic nevi and the least found is dermatofibroma. Skin diseases are taken from the different regions of the body. The regions are the back, lower extremity, trunk, upper extremity, abdomen, face, chest, foot, neck, scalp, hand, ear, genital, and sacral. Most patients are affected by skin disease in the back region and it is most prominent in men. Benign keratosis-like lesions are affected in the region face and Melanocytic nevi are affected in body parts of the patients except for the face. Melanocytic nevi diseases are the most prominent skin disease in the age group between 0–75. People aged 80–90 are affected more by Benign keratosis lesions. Skin disease in HAM10000 datasets is discovered by using histopathology (53.3%), follow-up examination (37.0%), expert consensus (9.0%), and confirmation by in-vivo confocal microscopy (0.7%). The samples were taken from different places in the patient’s body. This dataset is composed of 10,015 dermatoscopic images. The ground truth of the images is conducted by expert pathologists and medical experts. The images in the HAM10000 dataset were collected from the Department of Dermatology at the Medical University of Vienna, Austria, and Cliff Rosendahl in Queensland, Australia for a time period of around 20 years. Thus, the database is good and contains skin patient samples for various skin diseases. The images in HAM10000 are from seven different skin diseases. The detailed information on skin diseases and the technology involved in the database creation is discussed in detail by the authors [37].

**Figure 4:**
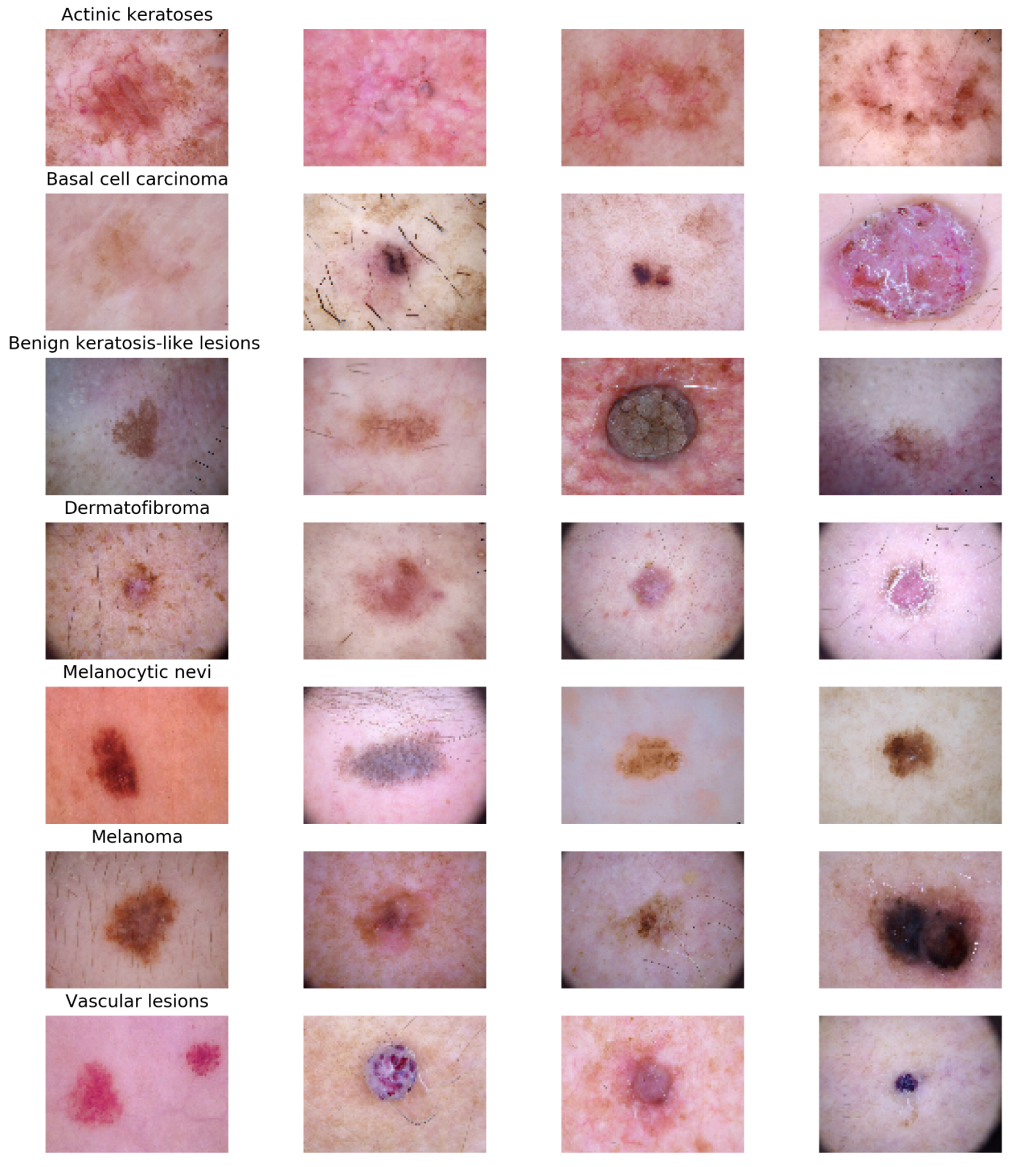
Skin disease samples of 7 classes in skin disease classification.

The samples of healthy and skin disease samples are shown in Figure 3. Skin disease samples of seven classes are shown in Figure 4. As shown in Figure 3 and Figure 4, the samples belonging to various classes in both datasets of skin disease detection, skin disease classification are similar and they have high intra-class and inter-class variance. In addition to that, the tiny affected region is important in accurately detecting and classifying the skin disease to its family of skin diseases. There may be a possibility that the CNN-based models might not give importance to these tiny regions. To avoid this, the current work integrates attention to the CNN architecture that can focus on the infected regions in the skin disease image.

## 5. Statistical Metrics

The proposed model for skin disease detection and skin disease classification is evaluated using the following statistical measures

Accuracy: The accuracy measure is estimated by dividing the total of correctly classified skin data samples by the total number of skin disease data samples. The accuracy metric gives equal importance to all the classes in the skin disease dataset. This may not be considered to be a good metric to evaluate the proposed model because the dataset used in skin disease classification is highly imbalanced.

(3) $$Accuracy=\frac{TP+TN}{TP+TN+FP+FN}$$

Precision: It is also called positive predictive value. It is the correct classification of the skin disease samples to the sum of the correct classification of the skin disease and incorrect classification of the skin disease in the given model. False positives should be less to get high precision.

(4) $$Precision=\frac{TP}{TP+FP}$$

Recall: It is also called sensitivity. It is the correct classification of the skin disease to the sum of the correct classification of the skin disease and the missed classification of the skin disease in the given model. False negative should be less to obtain high recall.

(5) $$Recall=\frac{TP}{TP+FN}$$

F1-score: It is the harmonic mean of precision and recall

(6) $$F1\;score=2\times \frac{Precision\times Recall}{Precision+Recall}$$

For a good skin disease detection and classification model, the precision, recall, and f1-score is close to 1.

TP, TN, FP, and FN denote true positive, true negative, false positive, and false negative, respectively, in accuracy, precision, recall, and f1-score. These are defined in the skin disease dataset, as given below

- TP: The number of skin disease data samples correctly predicted as skin disease.
- FN: The number of skin disease data samples wrongly predicted as normal.
- TN: The number of healthy patient data samples predicted as healthy.
- FP: The number of healthy patient data samples predicted as skin disease.

Using a confusion matrix, the TP, TN, FP, and FN are obtained. The confusion matrix counts the distribution of predictions across the actual labels of the skin disease dataset. The dimension of the confusion matrix is nXn, and *n* denotes the number of classes in the skin disease dataset. To estimate the performances of the proposed model at the class level in both skin disease detection and skin disease classification, precision, recall, and f1-score statistical metrics were considered in this work. The performances are reported for both the macro and weighted metrics of precision, recall, and f1-score. Macro measures are considered to be better for imbalanced datasets because the classes in the skin disease datasets are considered equally while computing the arithmetic mean of precision, recall, and f1-score of all the skin diseases. In the weighted metric, a support score is assigned while computing the arithmetic mean of precision, recall, and f1-score of all the skin diseases. The models are considered to be good if it shows high precision and high recall for rare skin diseases.

## 6. Results and Discussions

The experiments were conducted on the Kaggle GPU platform with hardware configurations: GPU P100 with 16 GB GPU memory, 13 GB CPU RAM, and 73.1 GB hard disk and libraries such as Keras, TensorFlow, scikit-learn with Python 3.5 for machine learning and deep learning model development.

**Figure 5:**
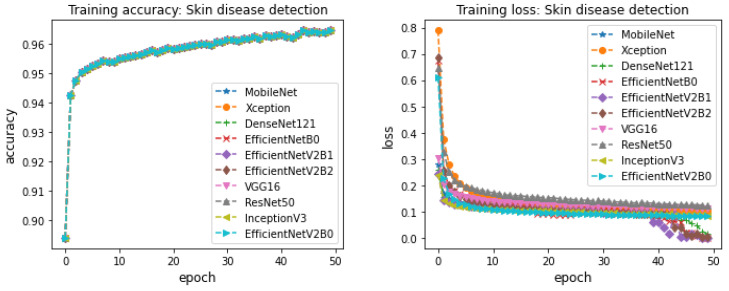
Training accuracy and training loss of CNN-based finetuned models for skin disease detection (left to right).

**Figure 6:**
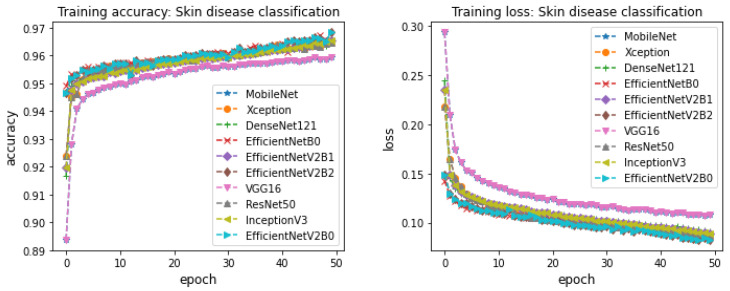
Training accuracy and training loss of CNN-based finetuned models for skin disease classification (left to right).

CNN-based pretrained models were trained on skin disease detection and skin disease classification. The CNN-based pretrained models considered in this work are Xception, VGG16, MobileNet, ResNet50, InceptionV3, DenseNet121, EfficientNetB0, EfficientNetV2B0, EfficientNetV2B1, and EfficientNetV2B2. These models contain several network parameters and network structures. The optimal performance depends on these network parameters and network structures. To find the best parameters for the network, various trials of experiments were run for the parameters’ optimizer, learning rate, epochs, and batch size. During training, the data samples in training and validation sets are shuffled to avoid bias in the training of a model. The optimal parameters for learning rate, epochs, batch size, and optimizer were 0.001, 50, 64, and adam, respectively. Various trials of experiments were run for optimizers such as adam, sgd, Adagrad, Adamax, and Nadam. The experiments with adam demonstrated successive improvement in training accuracy and validation accuracy and successive decrement in training loss and validation loss across epochs. Based on this, the optimizer parameter value is set to adam for the rest of the experiments. Next, to identify the optimal learning rate, the experiments were run for the learning rate in the range of 0.0001–0.5. The experiment with 0.001 demonstrated better training accuracy, validation accuracy, training loss, and validation loss during training. Thus, the learning rate is set to 0.001. To find the optimal parameters for batch size, the experiments were run for batches 32, 64, and 128. Due to limited access to memory, the batch size was not increased after 128. The experiments with 64 and 128 were almost similar in training accuracy and validation accuracy across epochs. Thus, the batch size is set to 64. Though batch size 128 slightly shows better performances for training accuracy and training loss, the batch size is set to 64. Because some of the CNN-based pretrained models result in memory issues, to find out the best parameter for epochs, the experiments were run for 70 epochs. However, all the models have not demonstrated any successive improvement in training accuracy and successive decrease in training loss after 50 epochs. Thus, we decided to set 50 epochs as optimal for the training of a model to detect skin diseases and classify them into the skin disease family. The training accuracy and training loss for the CNN-based pretrained models across 50 epochs for skin disease detection is shown in Figure 5. Figure 6 shows the training accuracy and training loss for the CNN-based pretrained models for skin disease classification. The models belonging to the EfficientNetV2 family demonstrated better accuracy by showing successive improvement in training accuracy and successive decrement in training loss compared to other CNN-based pretrained models in both skin disease detection and skin disease classification. Though the other models attained closer performance of training accuracy and training loss as EfficientNetV2, most of the models other than EfficientNetV2 did not show the same performance during testing. This is mainly due to the reason that most of the models other than EfficientNetV2 have reached the phase of overfitting and the models were not able to discriminate well among classes during testing for the new variants of skin disease images. VGG16 and MobileNet models demonstrated less performance in training accuracy and training loss compared to other CNN-based pretrained models in both skin disease detection and skin disease classification. Xception, DensNet121, ResNet50, and InceptionV3 models’ performance in terms of training accuracy and training loss were almost similar across epochs 50 but less compared to the models of a family of EfficientNet in both skin disease detection and skin disease classification. For skin disease detection, most of the models have demonstrated above 96% training accuracy and less than 0.1 training loss. EfficientNet models have reached above 99% training accuracy and less than 0.001 training loss for skin disease detection at the end of epochs 50. Most of the models have reached an accuracy of 95% and loss of less than 1 by epochs in the range of 15–20 for skin disease detection. The experiments were run to 50 epochs because the models have demonstrated successive improvement in training accuracy and training loss after 20 epochs. Similar performances were demonstrated by the CNN-based pretrained models for skin disease classification. In particular, the family of EfficientNet models has achieved 99% training accuracy with less than 0.01 training loss for skin disease classification. Most of the CNN-based models have demonstrated above 95% training accuracy and less than 0.1 training loss at epochs in the range 20–25. The experiments were continued until epoch 50 because the model has demonstrated a little successive improvement after epoch 25.

The total parameters for Xception, VGG16, MobileNet, ResNet50, InceptionV3, DenseNet121, EfficientNetB0, EfficientNetV2B0, EfficientNetV2B1, EfficientNetV2B2 are 23025711, 15306055, 4344519, 25751943, 23967015, 8153159, 5427363, 5427363, 7953031, and 9277433, respectively. The train parameters for Xception, VGG16, MobileNet, ResNet50, InceptionV3, DenseNet121, EfficientNetB0, EfficientNetV2B0, EfficientNetV2B1, fficientNetV2B2 are 22971183, 15306055, 4322631, 25698823, 23932583, 8069511, 5385347, 5385347, 7890983, and 9209865, respectively. The non-train parameters for Xception, VGG16, MobileNet, ResNet50, InceptionV3, DenseNet121, EfficientNetB0, EfficientNetV2B0, EfficientNetV2B1, fficientNetV2B2 are 54528, 0, 21888, 53120, 34432, 84648, 42016, 42016, 62048, and 67568, respectively. The trained models’ performances of CNN finetuned models for skin disease detection are reported in Table 4. Table 4 shows that the proposed model outperformed all the CNN-based pretrained models for skin disease detection with an accuracy of 99%. Results of the CNN-based models are reported in terms of Accuracy, weighted and macro precision, weighted and macro recall, and weighted and macro f1-score. The proposed model has improved the accuracy by 2% of the family of EfficientNetV2 models and 3% of the family of EfficientNet models. This shows that each CNN-based pretrained models learn its own feature representation and these features are unique. The proposed model takes advantage of the fusion of features of the family EfficientNetV2 models to accurately detect skin disease. Models such as ResNet50, InceptionV3, DenseNet121, and Xception demonstrated an accuracy of 92%, 93%, 93%, and 93%, respectively. These models performed lesser than the proposed model by accuracy in the range of 6–7%. In addition, the performances shown by ResNet50, InceptionV3, DenseNet121, and Xception on the test dataset for skin disease classification are lesser compared to the family of models of EfficientNet. Both the MobileNet and VGG16 demonstrated performances in terms of accuracy in the range of 88–89% for skin disease detection which is lesser than 10% accuracy compared to the proposed model. Overall, both MobileNet and VGG16 performed lesser than the proposed model, a family of EfficientNet models, and models such as ResNet50, InceptionV3, DenseNet121, and Xception. Along with accuracy, the performances for skin disease detection are reported in terms of macro and weighted precision, recall, and f1-score. The proposed model macro precision, macro recall, and macro f1-score is 99%, 99%, and 99%, respectively. Similar to the macro score, the proposed model showed 99% for weighted precision, weighted recall, and weighted f1-score. This indicates that the proposed model is effective in handling the imbalanced dataset. Macro and weighted performances of the proposed model are 2–3% higher compared to the family of EfficientNet models, 6–7% higher compared to the models such as ResNet50, InceptionV3, DenseNet121, and Xception, and 10–12% higher compared to the models such as VGG16 and MobileNet. Overall, the proposed method outperformed the existing CNN-based models and a family of EfficientNet models with better accuracy, precision, recall, and f1-score metrics for skin disease detection. The less performance in terms of accuracy, precision, recall, and f1-score are shown by the models such as MobileNet and VGG16.

For skin disease classification, the current work employed CNN-based pretrained models, and its results are reported in Table 5. The proposed approach outperformed all the other methods for skin disease classification in all the settings of test experiments. The table contains the results of the CNN-based pretrained models in terms of Accuracy, weighted and macro precision, weighted and macro recall, and weighted and macro F1-score. Since the dataset of skin disease classification is highly imbalanced, the work considers macro precision, macro recall, and macro f1-score. To demonstrate the differences between macro and weighted, this work reports the performances of all the models in both macro and weighted. As can be observed from the Table 5, the models show high precision, high recall, and high f1-score even though the misclassification rate is high in some rare skin diseases such as Dermatofibroma and Vascular lesions. The proposed model showed macro precision, macro recall, and macro f1-score of 97%, 100%, and 99%, respectively, and weighted precision, weighted recall, and weighted f1-score of 99%, 99%, and 99%, respectively. Though there is misclassification in the skin disease classes, the weighted measure shows 99% for precision, recall, and F1-score. However, macro precision, macro recall, and macro f1-score are considered to be best compared to weighted metrics because these metrics facilitate showing the individual class’s score with support instead of taking the average of all the classes. The family of EfficientNetV2 models showed better macro precision, macro recall, and macro f1-score over EfficientNetB0. The EfficientNetV2 models improved the macro precision, macro recall, and macro weighted metric of EfficientNetB0 model by 18%, 8%, and 13%, respectively. In a family of EfficientNetV2 models, EfficientNetV2B2 models outperformed the models EfficientNetV2B0 and EfficientNetV2B1 in all the settings of the experiments during testing a model for skin disease classification. The experiments are stopped at EfficientNetV2B2 models because there was no performance improvement by using other EfficientNetV2 models. Macro and weighted metrics of ResNet50, InceptionV3, and DenseNet121 models are in the range of 50–65%, 50–65% and 80–90%, respectively. These models’ performances are 30% lesser than the proposed model and a family of EfficientNet model compared to macro metrics and 20% lesser compared to weighted metrics of the proposed model and a family of EfficientNet models. Xception, VGG16, and MobileNet models showed macro precision, macro recall, and macro f1-score in the range of 40–60% and weighted precision, weighted recall, and weighted f1-score in the range of 80–90%. These model performances are almost 30% less compared to the macro metric of the proposed model and 10% less compared to the weighted metric of the proposed model. The proposed models showed an accuracy of 99% for skin disease classification by improving the accuracy in the range of 8–9% for the family of EfficientNet models. Models such as DenseNet121, InceptionV3, and ResNet50 showed an accuracy of 93%, 93%, and 92%, respectively, and their performances are lesser compared to the proposed model and the family of EfficientNet models. Similar to skin disease classification, models such as Xception, VGG16, and MobileNet showed an accuracy of 83%, 79%, and 76%, respectively. The model performances of Xception, VGG16, and MobileNet are lesser compared to the proposed model and other CNN-based pretrained models such as a family of EfficientNet models, DenseNet121, InceptionV3, and ResNet50. Overall, the proposed model showed better performances in accuracy and both macro and weighted metrics compared to the other CNN-based pretrained models. Since the proposed model has shown better performances in macro metrics compared to the existing CNN-based models on skin disease classification, the proposed model is considered to be effective in handling imbalanced skin disease datasets. Moreover, the proposed model is able to detect and classify rare skin diseases such as Vascular lesions and Dermatofibroma more accurately compared to other existing CNN-based pretrained models.

The detailed results of each class in skin disease detection and skin disease classification is reported in Table 6 and Table 7, respectively. In skin disease detection, the proposed approach demonstrated 99% accuracy, 99% precision, 99% recall, and 99% f1-score for both Healthy and Malignant classes. For skin disease classification, the proposed model showed 100% precision, 100% recall, and 100% f1-score for the skin diseases Actinic keratoses, Dermatofibroma, and Vascular lesions. For Melanoma and Melanocytic nevi, the proposed model demonstrated 96% precision, 100% recall, 98% f1-score, and 100% precision, 98% recall, and 99% f1-score, respectively. The proposed model for skin diseases such as Basal cell carcinoma and Benign keratosis-like showed 89% precision, 100% recall, 94% f1-score and 97% precision, 100% recall, and 99% f1-score, respectively. Overall, the proposed approach demonstrated better performance in all the classes in both skin disease detection and skin disease classification compared to other existing CNN-based pretrained models.

The confusion matrix for the CNN-based pretrained models for skin disease detection is included in Table 2. The proposed approach misclassification rate is 0.0111, which is lesser compared to all the other CNN-based pretrained models. The model misclassifies the three samples of Healthy as Malignant and five samples of Malignant as Healthy. The misclassification rate of EfficientNetB0, EfficientNetV2B0, EfficientNetV2B1, and EfficientNetV2B2 are 0.0401, 0.0333, 0.0292, and 0.0292, respectively. Models such as ResNet50, InceptionV3, Xception, and DenseNet121 showed misclassification rates of 0.0811, 0.0736, 0.0722, and 0.0722, respectively. The high misclassification rate is shown by models such as VGG16 and MobileNet. The misclassification rate of VGG16 and MobileNet are 0.1021 and 0.1153, respectively. All the models including the proposed approach demonstrated a high misclassification rate for the Malignant. This indicates that the model’s enhancement is required to avoid these misclassifications. There may be a possibility to avoid misclassification in the Malignant class by providing more data samples from different patients across different ages from different modalities. In addition to that, a detailed investigation and analysis of the proposed method needs to be analyzed to understand the misclassification. The optimal features that are used to accurately detect the Malignant data samples need to be analyzed in detail to understand the misclassification. This type of study of the proposed model can be considered as one of the significant directions toward future work.

**Figure 7:**
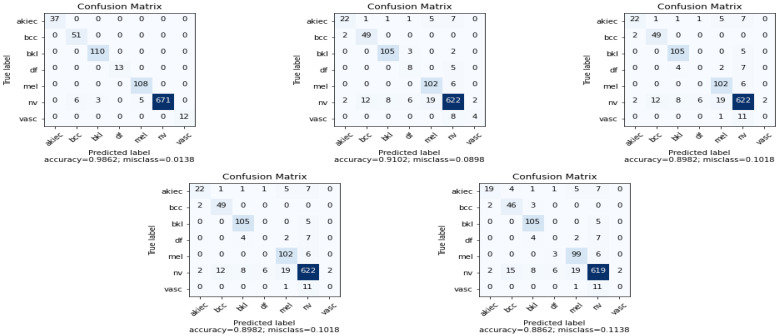
Skin disease classification confusion matrix using Proposed Model, EfficientNetB0, ResNet50, InceptionV3, and DenseNet121 (left to right).

The confusion matrix for the CNN-based pretrained model for skin disease classification is shown in Figure 7. The proposed model showed a 0.0138 misclassification rate, which is less compared to all the CNN-based pretrained models. The misclassification rate of EfficientNetB0, EfficientNetV2B0, EfficientNetV2B1, and EfficientNetV2B2 are 0.09, 0.07, 0.06, and 0.04, respectively. Models such as ResNet50, InceptionV3, and DenseNet121 showed a high misclassification rate compared to the models of a family of EfficientNet. The high misclassification shown by the models Xception, VGG16, and MobileNet is compared to all the other CNN-based pretrained models in skin disease classification. The proposed approach classified all the samples of Actinic keratoses, Basal cell carcinoma, Benign keratosis-like lesions, Dermatofibroma, Melanoma, and Vascular lesions. For Melanocytic nevi, the proposed model misclassified six samples as Basal cell carcinoma, three samples as Benign keratosis-like lesions, and five samples as Melanoma. Most importantly, except the proposed models, the other existing models have shown a high misclassification rate for rare skin diseases such as Vascular lesions and Dermatofibroma. The models such as ResNet50, InceptionV3, VGG16, MobileNet, Xception, and DenseNet121 have failed to classify a single sample correctly for Vascular lesions and Dermatofibroma. This indicates that these models are not effective in highly imbalanced skin disease datasets. Though these models are effective in other classes and demonstrated accuracy above 85%, the existing models are not effective for rare skin diseases in both skin disease detection and skin disease classification. In addition to rare skin diseases, the models other than the proposed approach and a family of EfficientNet showed a high misclassification rate. Since the proposed approach is a fused model of a family of EfficientNetV2, it outperformed a single-finetuned EfficientNetV2 model and EfficientNet model in both skin disease detection and skin disease classification. Overall, the proposed model has demonstrated less misclassification rate for all the classes in skin disease classification compared to other CNN-based pretrained models.

The proposed model has outperformed the CNN-based pretrained models in all the test settings in both skin disease detection and skin disease classification. Most importantly, the proposed model integrates the cost weight to the deep learning model, which helped to demonstrate a better classification rate compared to the existing approaches. In addition to that, the proposal of a meta-classifier in the final classification helped to achieve generalization and to make the model to be more robust in detecting the skin disease and classifying the skin disease to its family. Since the proposed model is an ensemble of various EfficientNetV2 models, the model has learned better feature representation to accurately detect and classify skin diseases. The result of the ensemble feature representation of pretrained model has performed better than the single CNN-based pretrained model. In the proposed work, the CNN-based pretrained model and the classification model are not integrated together during training in skin disease detection and the skin disease classification model. Thus, proposing a loss function to integrate the CNN-based pretrained model and classification model will be considered one of the significant directions toward future work. The proposed model employs a KPCA-based dimensionality reduction to reduce the features of CNN-based pretrained models. There may be a possibility that the loss of features can happen in this stage. Detailed analysis and experiments can be demonstrated using the different dimensions of features for skin disease detection and classification. This type of experimental work can be considered future work. The proposed model is a hybrid of CNN-based pretrained models and meta-classifiers. The detailed model parameters of the CNN-based pretrained models for skin disease detection and classification are reported. However, discussion of algorithm complexity analysis to the experimental part in skin disease detection and classification is important and this will be another direction towards future works.

## 7. Conclusions and Future Works

This paper proposed an attention-cost-sensitive deep learning-based feature fusion ensemble meta-classifier approach for skin cancer detection and classification. The proposed model integrates attention to the deep learning model to detect the infected tiny regions of the overall skin image. To give importance to the minority classes during the training, cost-weights were introduced. The proposed work assigns higher class weights to the classes that have fewer skin disease data samples and lower-class weights to the classes that have a high number of skin disease data samples. The proposed model fuses the features of EfficientNetV2 pretrained models, and the dimensionality reduction of the features is conducted using KPCA. Further, the reduced features are passed into ensemble meta-classifiers. In the first stage of the ensemble meta-classifiers, the prediction of the skin disease is conducted using RFTree and SVM, and the logistic regression performs the classification by considering the probability of the first-stage classifiers as features. In all the experimental settings of the proposed model, the proposed model outperformed the existing methods for both skin cancer detection and skin cancer classification. The proposed model improves the accuracy by 4% compared to the existing approaches for skin disease detection and 9% for skin disease classification. Since clinical features play an important role in enhancing the detection rate of skin diseases, the clinical features of skin diseases can be included along with skin images. This type of fused features of clinical and non-clinical can improve the performance of the model in accurately detecting the skin disease and classifying them into their disease family. The deep learning models are not robust in an adversarial environment and there may be a possibility that the deep learning models can be bypassed by following the techniques available in the field of adversarial machine learning. Thus, the detailed evaluation of the proposed model to detect and classify skin diseases in an adversarial environment will be considered as future work.

## Figures and Tables

**Table 1:** Summary of existing methods for skin disease classification.

Reference	Methodology	Dataset	Pretrained Model	Effective for Class Imbalance	Accuracy	Attention	Large- Scale Learning	Feature Fusion
[9]	GoogleNet Inception v3	ISIC Archive	Yes	No	93.3%	No	No	No
[10]	ResNet50	ISIC Archive	Yes	No	82%	No	No	No
[11]	CNN	ISIC 2017	Yes	No	83%	No	No	No
[12]	ResNet-50	ISIC 2018	Yes	No	94%	No	No	Yes
[13]	ResNet-152	HAM10000	Yes	No	92%	No	No	No
[14]	SE-Resnext50	HAM10000	Yes	Yes	-	Yes	No	Yes
[15]	CNN	ISIC Archive	No	No	97.49	No	No	No
[16]	GoogLeNet Inception-v3	ISIC 2018	Yes	No	67–73%	No	No	No
[36]	VGG, ResNet, AlexNet	ISIC 2019	Yes	No	95%	No	No	No
[17]	VGG, ResNET-50, Inception, MobileNet, DenseNet, Xception	ISIC 2018	Yes	No	92.4	No	Yes	Yes
[18]	Inception, ResNet	ISIC 2016, 2017, and 2018	Yes	No	81.79	No	No	No
[19]	EfficientNet	ISIC 2016, ISIC 2017	Yes	No	86.2%	No	No	Yes
[20]	ResNet	ISIC 2018	Yes	No	95.2	Yes	No	No
[21]	ResNet	Private dataset	Yes	No	79%	No	No	No
[22]	EfficientNet, ResNet	ISIC 2019	Yes	No	-	No	No	No
[23]	CNN and Naïve Bayes	ISIC Archive	No	No	93.6%	No	Yes	No
[24]	VGG ResNet DenseNet	ISIC 2018	Yes	Yes	87.06%	No	Yes	Yes
[25]	ResNeXt101	HAM 10000	Yes	No	92.83%	No	No	No
[26]	ResNet	HAM 10000	Yes	No	80–90%	No	No	No
[27]	GoogleNet, ResNet-101, & NasNet-Large	ISIC 2019	Yes	No	89%	No	No	Yes
[28]	Inception	Private dataset	Yes	No	70–75%	No	No	No
[35]	CNN	Private dataset	No	No	-	No	No	No
[29]	ResNet152 and InceptionResNet-V2	Private dataset	Yes	No	87.42	No	No	No
[30]	CNN	ISIC 2017 ISIC 2018 ISIC 2019	No	No	85–90%	No	No	No
[31]	ResNet	ISIC 2018	Yes	Yes	70.1%	Yes	No	No
[32]	DenseNet	ISBI 2016 ISBI 2017 HAM10000	Yes	No	93.6%	No	Yes	No
[33]	DenseNet	HAM10000	Yes	No	96.18	No	No	No
[34]	MobileNet	HAM10000	Yes	No	85%	No	Yes	No
Proposed	EfficientNetV2B0, EfficientNetV2B1, & EfficientNetV2B2	HAM10000, ISIC Archive	Yes	Yes	99%	Yes	Yes	Yes

**Table 2:** Statistics of skin disease detection dataset.

Class	Training	Testing	Total
Healthy	1440	360	1800
Malignant or Skin Cancer	1197	360	1557
Total	2637	720	3357

**Table 3:** Statistics of skin disease classification dataset.

Class	Training	Testing	Total
Melanocytic nevi (nv)	6034	671	6705
Melanoma (mel)	1005	108	1113
Benign keratosis-like lesions (bkl)	989	110	1099
Basal cell carcinoma (bcc)	463	51	514
Actinic keratoses (akiec)	290	37	327
Vascular lesions (vasc)	130	12	142
Dermatofibroma (df)	102	13	115
Total	9013	1002	10,015

**Table 4:** Detailed results for skin disease detection.

Model	Accuracy	Type	Precision	Recall	F1-Score	Confusion Matrix
Xception	0.93	Macro	0.93	0.93	0.93	[342 18] [34 326]
		Weighted	0.93	0.93	0.93	
VGG16	0.89	Macro	0.89	0.89	0.89	[333 27] [50 310]
		Weighted	0.89	0.89	0.89	
MobileNet	0.88	Macro	0.89	0.88	0.88	[330 30] [53 307]
		Weighted	0.89	0.88	0.88	
ResNet50	0.92	Macro	0.92	0.92	0.92	[336 24] [36 324]
		Weighted	0.92	0.92	0.92	
InceptionV3	0.93	Macro	0.93	0.93	0.93	[338 22] [31 329]
		Weighted	0.93	0.93	0.93	
DenseNet121	0.93	Macro	0.93	0.93	0.93	[340 20] [32 328]
		Weighted	0.93	0.93	0.93	
EfficientNetB0	0.96	Macro	0.96	0.96	0.96	[350 10] [19 341]
		Weighted	0.96	0.96	0.96	
EfficientNetV2B0	0.97	Macro	0.97	0.97	0.97	[352 8] [16 344]
		Weighted	0.97	0.97	0.97	
EfficientNetV2B1	0.97	Macro	0.97	0.97	0.97	[355 5] [16 344]
		Weighted	0.97	0.97	0.97	
EfficientNetV2B2	0.97	Macro	0.97	0.97	0.97	[355 5] [16 344]
		Weighted	0.97	0.97	0.97	
Proposed	0.99	Macro	0.99	0.99	0.99	[357 3] [5 355]
		Weighted	0.99	0.99	0.99	

**Table 5:** Detailed results for skin disease classification.

Model	Accuracy	Type	Precision	Recall	F1-Score
Xception	0.83	Macro	0.57	0.59	0.58
		Weighted	0.84	0.83	0.84
VGG16	0.79	Macro	0.50	0.51	0.50
		Weighted	0.80	0.79	0.80
MobileNet	0.76	Macro	0.46	0.44	0.44
		Weighted	0.78	0.76	0.77
ResNet50	0.85	Macro	0.56	0.55	0.54
		Weighted	0.86	0.85	0.85
InceptionV3	0.89	Macro	0.59	0.60	0.59
		Weighted	0.88	0.89	0.88
DenseNet121	0.90	Macro	0.61	0.63	0.61
		Weighted	0.89	0.90	0.89
EfficientNetB0	0.91	Macro	0.78	0.76	0.75
		Weighted	0.91	0.91	0.91
EfficientNetV2B0	0.93	Macro	0.82	0.83	0.82
		Weighted	0.93	0.93	0.93
EfficientNetV2B1	0.94	Macro	0.91	0.84	0.86
		Weighted	0.95	0.94	0.94
EfficientNetV2B2	0.96	Macro	0.96	0.84	0.88
		Weighted	0.96	0.96	0.96
Proposed	0.99	Macro	0.97	1.00	0.99
		Weighted	0.99	0.99	0.99

**Table 6:** Detailed results of Healthy and Malignant classes in skin disease detection.

Class	Precision	Recall	F1-Score
Healthy	0.99	0.99	0.99
Malignant	0.99	0.99	0.99
accuracy	0.99		
macro avg	0.99	0.99	0.99
weighted avg	0.99	0.99	0.99

**Table 7:** Detailed results of each classes in skin disease classification.

Class	Precision	Recall	F1-Score
Actinic keratoses (akiec)	1.00	1.00	1.00
Basal cell carcinoma (bcc)	0.89	1.00	0.94
Benign keratosis-like lesions (bkl)	0.97	1.00	0.99
Dermatofibroma (df)	1.00	1.00	1.00
Melanoma (mel)	0.96	1.00	0.98
Melanocytic nevi (nv)	1.00	0.98	0.99
Vascular lesions (vasc)	1.00	1.00	1.00
accuracy	0.99		
macro avg	0.97	1.00	0.99
weighted avg	0.99	0.99	0.99

## Data Availability

Data available on request from the author.
